# The C2 Protein from the Geminivirus *Tomato Yellow Leaf Curl Sardinia Virus* Decreases Sensitivity to Jasmonates and Suppresses Jasmonate-Mediated Defences

**DOI:** 10.3390/plants5010008

**Published:** 2016-01-15

**Authors:** Tábata Rosas-Díaz, Alberto P. Macho, Carmen R. Beuzón, Rosa Lozano-Durán, Eduardo R. Bejarano

**Affiliations:** 1 Departamento de Biología Celular, Genética y Fisiología, Instituto de Hortofruticultura Subtropical y Mediterránea, Universidad de Málaga—Consejo Superior de Investigaciones Científicas, Campus de Teatinos, E-29071 Malaga, Spain; tabatarosas@sibs.ac.cn (T.R.-D.); alberto.macho@sibs.ac.cn (A.P.M.); cbl@uma.es (C.R.B.); 2 Shanghai Center for Plant Stress Biology (PSC), Shanghai Institutes of Biological Sciences, Chinese Academy of Sciences, Shanghai 201602, China

**Keywords:** plant virus, jasmonates, defence, C2, SCF^COI1^, coronatine, geminivirus

## Abstract

An increasing body of evidence points at a role of the plant hormones jasmonates (JAs) in determining the outcome of plant-virus interactions. Geminiviruses, small DNA viruses infecting a wide range of plant species worldwide, encode a multifunctional protein, C2, which is essential for full pathogenicity. The C2 protein has been shown to suppress the JA response, although the current view on the extent of this effect and the underlying molecular mechanisms is incomplete. In this work, we use a combination of exogenous hormone treatments, microarray analysis, and pathogen infections to analyze, in detail, the suppression of the JA response exerted by C2. Our results indicate that C2 specifically affects certain JA-induced responses, namely defence and secondary metabolism, and show that plants expressing C2 are more susceptible to pathogen attack. We propose a model in which C2 might interfere with the JA response at several levels.

## 1. Introduction

Geminiviruses are a large family of plant viruses with circular, single-stranded (ss) DNA genomes packaged within geminate particles [1], which infect a broad range of staple and fiber crops worldwide and cause devastating diseases that lead to serious economic losses. Geminiviral genomes are small, ranging from 2.5 to 5.5 Kb, which imposes limitations in coding capacity. These viruses, however, seem to have compensated these restrictions by evolving overlapping and bidirectional open reading frames (ORFs), encoding four to eight multifunctional proteins that effectively manipulate plant functions to favor infection.

The multifunctionality of geminiviral proteins can be exemplified by C2 from monopartite geminiviruses belonging to the genus Begomovirus. Begomoviral C2 is a small protein, around 15 KDa in size, which localizes mainly in the nucleus of the plant cell. This protein has been shown to be required for either viral infection or full infectivity in several cases, suggesting a high-value role during geminivirus infection [2,3,4,5,6]. C2 has been described as a transcription factor for viral genes [7,8] and a suppressor of gene silencing, both post-transcriptional (PTGS) and transcriptional (TGS) [2,9,10,11,12,13,14,15]. Additionally, C2 from two different begomoviruses, *Tomato yellow leaf curl Sardinia virus* (TYLCSV) and *Tomato yellow leaf curl virus* (TYLCV), interacts with the catalytic subunit of the CSN (COP9 signalosome) complex, affecting the ability of the CSN to regulate ubiquitin E3 ligase complexes belonging to the SCF (SKP1, CUL1/CDC53, F-box proteins) family [4]. Consequently, C2 impairs cellular processes regulated by SCF complexes when transgenically expressed in *Arabidopsis*; remarkably, the response to jasmonates specifically appears significantly repressed by C2 in microarray analyses [4].

The oxylipin jasmonic acid (JA) and its metabolites, collectively known as jasmonates (JAs), are important plant signalling molecules that mediate biotic and abiotic stress responses, as well as several aspects of plant growth and development [16]. In basal conditions, JA levels are low and JA-mediated transcriptional responses are kept in a repressed state by JASMONATE ZIM-DOMAIN (JAZ) proteins. In response to stresses, such as insect feeding or necrotrophic pathogen infection, an increase in the levels of the bioactive jasmonate JA-Ile allows this hormone to act as molecular glue, facilitating the interaction between the JAZ repressors and the F-box protein CORONATINE INSENSITIVE 1 (COI1), recognition component of the JA receptor, the E3 ubiquitin ligase SCF^COI1^. The result of this interaction is the targeting of JAZs for ubiquitination and degradation via the 26S proteasome pathway, allowing for the expression of JA-responsive genes [17,18]. JA-dependent transcriptional reprogramming is regulated by a cascade of transcription factors (TFs), in which MYC2 plays a major role, as indicated by the lower sensitivity to JA displayed by the *jin1* mutant, carrying a mutation in the *MYC2* gene [19,20]. JAZ proteins directly interact with MYC2 in the absence of JA, keeping this transcription factor inactive [17]; degradation of JAZ proteins in response to JAs allows MYC2 to exert its effect on downstream target genes. JAZ proteins have also been shown to interact with MYC3 and MYC4, among other TFs [16,21,22,23]. Notably, JAZ expression is induced after JA perception or wounding, indicating that JAZ repressors are also JA-responsive genes, as part of a negative feedback loop regulation of JA responses [24].

Although traditionally jasmonate-mediated defences have been ascribed a role against necrotrophic pathogens and herbivorous insects, a growing body of evidence now points at these hormones as acting also in plant-virus interactions [17,25,26,27,28,29,30,31,32] Moreover, interference with JA-regulated gene expression seems to be a general property of viral suppressors or RNA silencing, since it has been observed in transgenic plants expressing p25, HC-Pro, 126 KDa and 2b [33], as well as geminiviral C2 [4], V2 (Luna, Lozano-Durán and Bejarano, unpublished), and βC1 [31,34].

Recent findings indicate that jasmonate signalling is also altered by geminiviruses. Repression of the jasmonate pathway or jasmonate-responsive genes have been reported in transgenic plants expressing a pathogenicity factor encoded by the DNAβ of *Tomato yellow leaf curl China virus* (TYLCCNV) βC1, C2 from TYLCV or TYLCSV, and in *Arabidopsis* plants infected with *Cabbage leaf curl virus* (CaLCuV) [4,31,34,35]. Furthermore, exogenous application of methyl jasmonate (MeJA) interferes with *Beet curly top virus* (BCTV) infection in *Arabidopsis*, leading to milder symptoms and lower viral accumulation [4]. Remarkably, expression of jasmonic acid biosynthetic genes has been associated to the recovery process in geminivirus-infected pepper [36].

The transcriptional inhibition of the jasmonate signalling pathway and the decreased JA responses in plants expressing C2 from TYLCV or TYLCSV [4] may be linked to an impairment of the function of the jasmonate receptor, the SCF^COI1^ complex, given the effect of these proteins on the CSN complex and the SCF ubiquitin E3 ligases, in general. However, both the exact molecular mechanisms underlying this effect and its extent remain to be determined. In this work, we analyze, in detail, the suppression of the JA response exerted by C2 using transcriptomic analyses, as well as the effect of C2 on plant defence through pathogen challenge of C2 transgenic plants. Strikingly, C2-expressing plants show a suppression of JA-mediated defence processes as well as JA-dependent secondary metabolism, which may involve additional, specific protein-protein interactions.

## 2. Results

### 2.1. Transgenic Arabidopsis Plants Expressing C2 from TYLCV or TYLCSV Are Less Sensitive to Bacterial Coronatine

**Figure A1:**
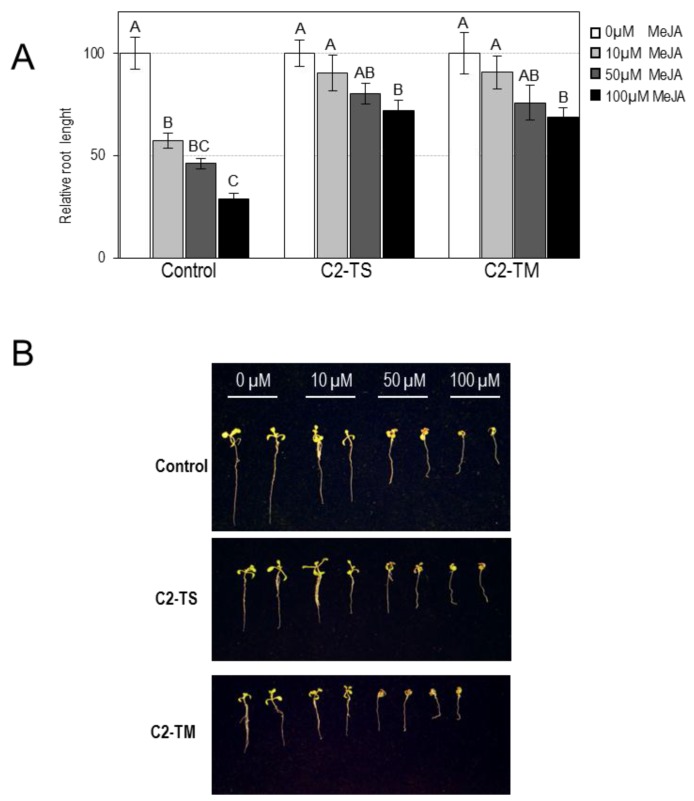
Root growth inhibition assays in C2-expressing plants. (**A**) Relative root length of wild-type Col-0 (Control), C2-TS (plants expressing the *C2* gene from TYLCSV), and C2-TM (plants expressing the *C2* gene from TYLCV) *Arabidopsis* seedlings in increasing concentrations of MeJA (0, 10, 50, and 100 μM). The values are the mean of at least ten seedlings. Bars represent standard error. One-way ANOVA Tukey’s Multiple comparison tests were used to distinguish differences among samples at *p*-value < 0.05. Different letters indicate statistically significant difference. Experiments were repeated three times with similar results; results from one representative experiment are shown; (**B**) Pictures of representative seedlings used in (**A**).

As a first step to further understand the effect of C2 on the plant response to JAs, we carried out a root growth inhibition assay in increasing concentrations of methyl-jasmonate (MeJA). As shown in Figure A1, consistent with our previous observations, the MeJA-induced inhibition of root growth is significantly less pronounced in *Arabidopsis* lines expressing C2 than in control plants for all hormone concentrations tested.

**Figure 1:**
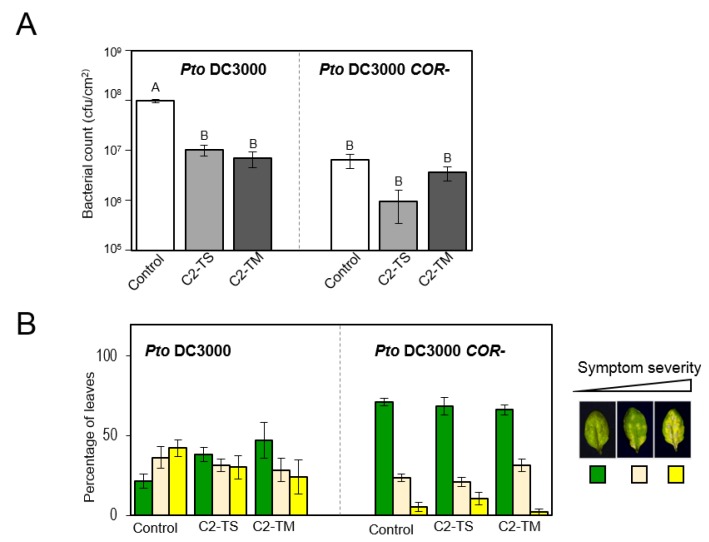
Infection of C2-expressing plants with *Pseudomonas syringae* pv. *tomato* DC3000. (**A**) Bacterial growth of wild-type (*Pto* DC3000) or a deficient strain unable to synthesize coronatine (COR-) in wild-type Col-0 (control) or transgenic C2-expressing plants in dip-inoculation experiments. Samples were taken at 4 dpi. Values are the mean of five plants. Bars represent standard error. One-way ANOVA Tukey’s Multiple comparison tests were used to distinguish differences among samples at *p*-value <0.05. Different letters indicate statistically significant difference. Results are the mean of three independent biological replicates; (**B**) Symptoms displayed by dip-inoculated plants. Three different categories are considered: no symptoms, few symptoms or full symptoms, as indicated in the legend. The percentage of leaves in each category is represented. Bars represent standard error. In both (**A**) and (**B**), experiments were repeated three times with similar results; results from one representative experiment are shown.

Given that C2 has been shown to affect the function of several SCF complexes in the plant [4], it would be feasible to speculate that the lower sensitivity to jasmonates displayed by the transgenic C2 plants could be due to a malfunction of the SCF^COI1^ complex. This SCF complex acts as the jasmonate receptor, but is also the receptor for the bacterial toxin coronatine, an analogue of the bioactive jasmonate JA-Ile which is synthesized and secreted by the plant pathogenic bacterial strain *Pseudomonas syringae* pv. *tomato* DC3000 (*Pto* DC3000) [37,38,39]. Consequently, if the activity of the SCF^COI1^ is hindered in the presence of the viral protein, transgenic C2 plants should also be less sensitive to coronatine. In order to test this, transgenic C2 plants were dip-inoculated with *Pseudomonas syringae* pv. *tomato* (*Pto*) DC3000 wild-type or a mutant unable to synthesize coronatine (COR-) and bacterial growth was measured four days post-inoculation (dpi). In the dipping inoculation method, bacteria are forced to enter the plant tissues through natural openings, such as stomata. However, following perception of pathogen-associated molecular patterns (PAMPs), stomatal closure is triggered to prevent pathogen entry; this closure, nevertheless, can be reverted by the successful pathogen *Pto* DC3000 through the activity of coronatine inside the plant cell [38]. Therefore, wild-type bacteria will trigger the re-opening of the stomata after toxin production and its perception by the plant SCF^COI1^ complex, allowing bacterial entry, whereas coronatine-defective bacteria will not, and will thus invade the plant tissues less efficiently [38]. Consistently with this model, our results show that growth of the COR- mutant when dip-inoculated into wild-type *Arabidopsis* plants is reduced compared to growth of wild-type bacteria (Figure 1A). However, this reduction is lower in the plants expressing C2 from TYLCSV and absent in plants expressing C2 from TYLCV, since bacterial numbers are similar for both strains regardless of whether they can produce coronatine, and similar to those obtained for the COR- mutant in wild-type plants (Figure 1A). A good correlation can be found between symptom severity and bacterial numbers (Figure 1B). These results indicate that the coronatine toxin produced by wild-type bacteria is not properly exerting its function in the C2 transgenic plants. Although we cannot formally rule out that additional targets of C2 impact susceptibility to *P. syringae*, the small difference between wild-type and coronatine-deficient bacteria in C2 plants can be more likely explained by a combination of two observations: (i) the activity of the SCF complexes is not completely impaired in the C2 plants, but rather partially hindered, so the toxin produced by wild-type bacteria is expected to exert some activity; and (ii) in the absence of coronatine, bacteria will only be able to enter the plant tissues through open stomata whose PAMP-triggered closure has not been accomplished yet. In the C2 plants, stomata are more efficiently closed as a consequence of ABA hypersensitivity of the guard cells [4], so bacterial entry will be further hampered. Our results are consistent with a reduced sensitivity to coronatine displayed by the C2 plants and, together with the lower sensitivity to exogenously applied MeJA, support a malfunction of the SCF^COI1^ complex.

### 2.2. C2 Represses Transcriptional JA Responses and JA-Induced Defences

**Figure 2:**
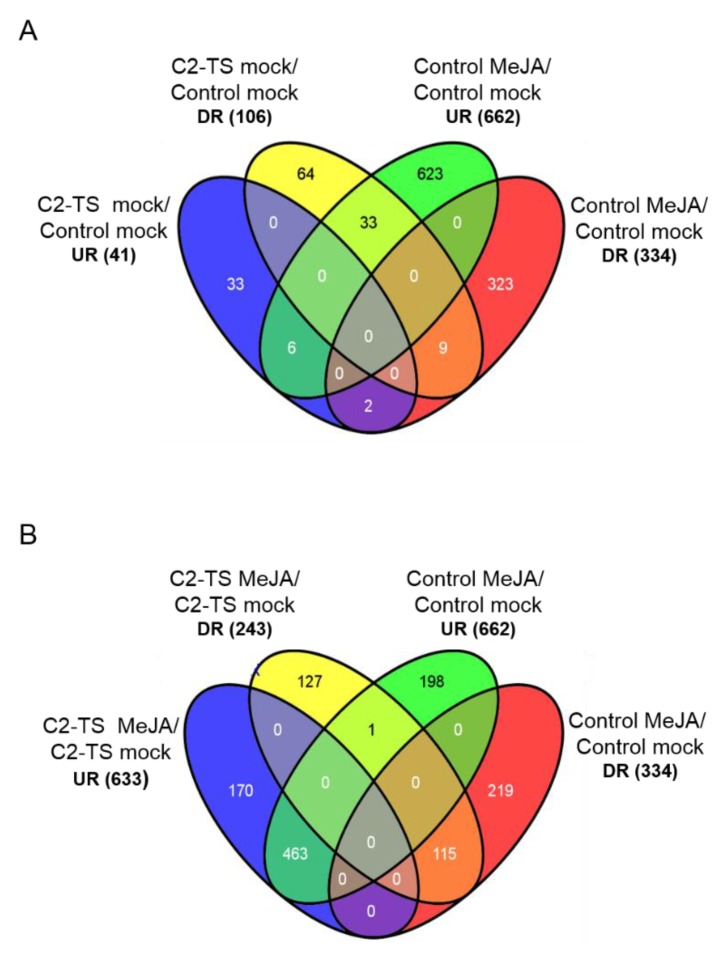
Venn diagrams showing the number of genes up- or down-regulated (UR or DR, respectively) in C2-TS plants, either JA- or mock-treated (in (**A**,**B**), respectively), and JA-treated control plants. The Venn diagrams were constructed using the software Venny (http://bioinfogp.cnb.csic.es/tools/venny). Total number of genes in each subset is indicated in brackets.

**Figure A2:**
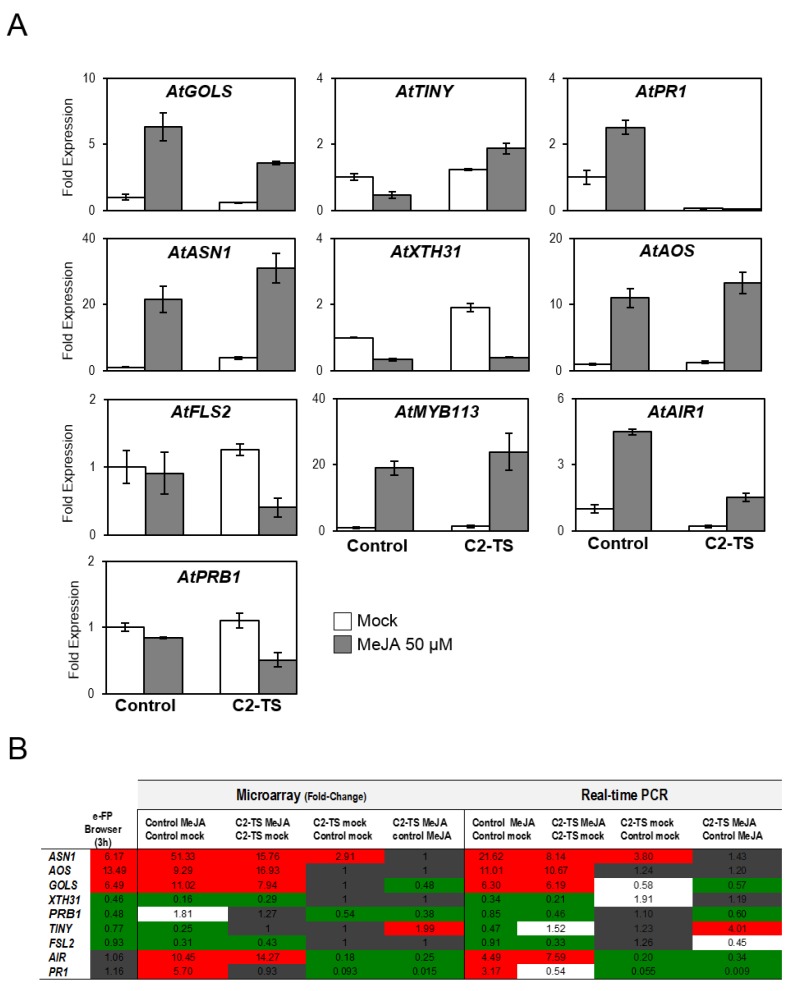
Expression levels of JA-regulated and control genes in Arabidopsis transgenic C2-TS and control plants for microarray validation. (**A**) Relative expression level of *AtASN1* (At3g47340), *AtAOS1* (At5g42650), *AtGOLS* (At2g47180), *AtXTH31* (At3g44990), *AtPRB1* (At2g14580), *AtTINY* (At5g25810), *AtFLS2*; (At5g46330), *AtAIR1* (At4g12550) and *AtPR1* (At2g14610) genes in transgenic C2-TS and control Arabidopsis seedlings, mock- or MeJA-treated, determined by real-time PCR. Values are the mean of three technical replicates. Bars represent standard error; (**B**) Comparison between microarray and real-time PCR expression data of the MeJA-regulated and control genes in Arabidopsis transgenic C2-TS and control plants used in (**A**). Up- and down-regulation are shown in red and green, respectively; lack of differential expression or coincidence between the two methods are shown in grey and white, respectively. Values from eFP browser after 3 h of JA treatment are also represented [52] (Arabidopsis eFP Browser. Available online: http://bar.utoronto.ca/efp/cgi-bin/efpWeb.cgi). The numbers represent the expression value in fold change.

With the aim of gaining insight into the effect of C2 on the response to jasmonates, we did a microarray analysis of *Arabidopsis* transgenic plants expressing C2 from TYLCSV (C2-TS plants) in both basal conditions and after MeJA treatment. For the analysis of transcriptomic data, four comparisons were carried out: (i) C2-TS *versus* control plants (mock-treated); (ii) MeJA-treated control plants *versus* mock-treated control plants; (iii) MeJA-treated C2-TS plants *versus* mock-treated C2-TS plants; and (iv) MeJA-treated C2-TS plants *versus* MeJA-treated control plants. Differential expression of selected genes was validated by quantitative real-time PCR (Figure A2). The number of up- and down-regulated genes in each comparison is represented in Figure 2. The expression of C2 in basal conditions causes transcriptional changes, especially involving down-regulation of gene expression, similar to previous results using a CATMA microarray [4]. MeJA treatment triggers dramatic transcriptional changes in *Arabidopsis*, similar to those shown in previous works [40,41]. Transcriptional changes observed in MeJA-treated C2-TS transgenic plants reveal a reduced response to the hormone: the number of either up- or down-regulated genes after MeJA treatment is lower in the C2-TS plants, and comparison between MeJA-treated C2-TS and control plants reveals a subset of genes differentially expressed in response to MeJA in the presence of C2, most of them being down-regulated, which indicates the existence of a group of jasmonate-responsive genes that do not respond or respond to a lower extent in the C2-TS plants (Figure 2).

**Figure 3:**
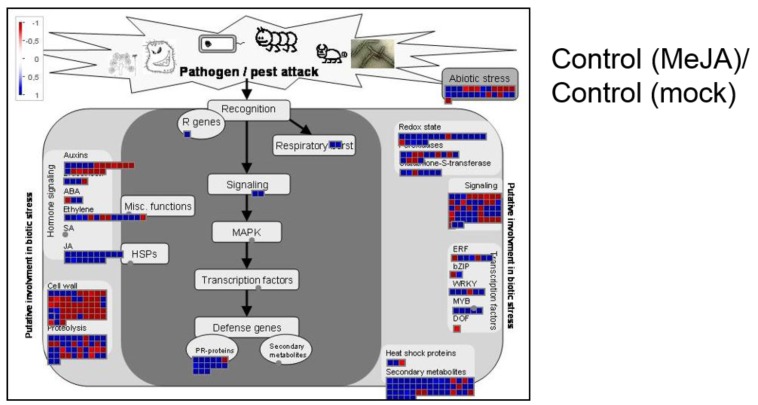

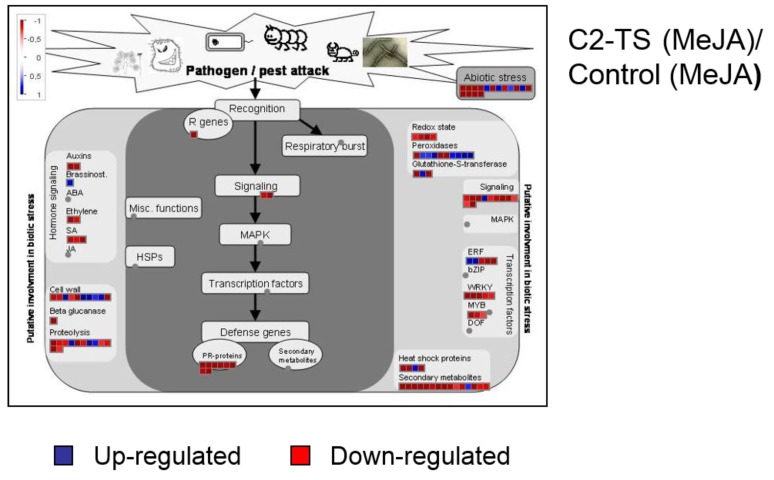
MapMan visualization of defence-related differentially expressed genes in response to MeJA in control or C2-TS-expressing plants.

In order to explore which functional categories are affected by expression of C2 and/or MeJA treatment, a GO functional enrichment analysis was performed using the VirtualPlant BioMaps tool ([42]; http://virtualplant.bio.nyu.edu/cgi-bin/vpweb/). Table 1, Table 2 and Table 3 show the non-redundant GO terms over-represented in the following subsets of genes: JA-responsive genes repressed by C2 in basal conditions (Table 1; 33 genes); genes which are JA-responsive in control plants only (Table 2; 198 up-regulated and 219 down-regulated genes); and genes differentially expressed in C2 plants in response to JA, as compared to control plants (Table 3). Our results indicate that the response to JA in plants expressing C2 is both qualitatively and quantitatively different to that in wild-type plants, and that the main JA-regulated responses affected by C2 are defence responses and secondary metabolism. A MapMan representation of the effect of C2 on JA-induced defence responses is depicted in Figure 3.

**Table 1:** Over-represented GO categories (biological function ontology) in the subset of JA-responsive genes repressed by C2 in basal conditions.

JA-Responsive Genes Repressed by C2
Response to JA
Response to biotic stimulus
Response to wounding
Lipid transport

**Table 2:** Over-represented GO categories (biological function ontology) in the subset of JA-responsive genes in control plants only.

JA-Responsive Genes in Control Plants Only
*Up-regulated genes*
Response to stress
Response to JA
Response to wounding
Defence response
Biotic stimulus
Secondary metabolism
*Down-regulated genes*
Growth
Cell wall organization
Response to auxin and gibberellin
Lipid metabolism and transport

**Table 3:** Over-represented GO categories (biological function ontology) in the subset of differentially-expressed genes in C2-expressing plants in response to JA, as compared to control plants.

Differentially Expressed Genes in C2 Plants in Response to JA (Compared to Control Plants)
*Up-regulated genes*
Response to stress
Response to oxidative stress
*Down-regulated genes*
Defence response
Multi-organism process
Immune system
Secondary metabolism
Lipid transport

### 2.3. Transgenic Plants Expressing C2 Are More Susceptible to an RNA Virus and a Plant-Pathogenic Bacterial Strain

**Figure 4:**
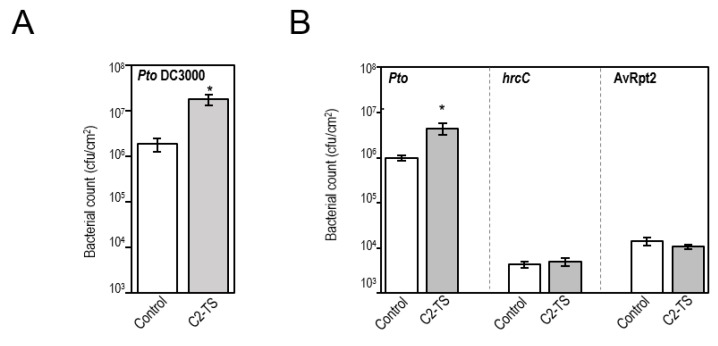

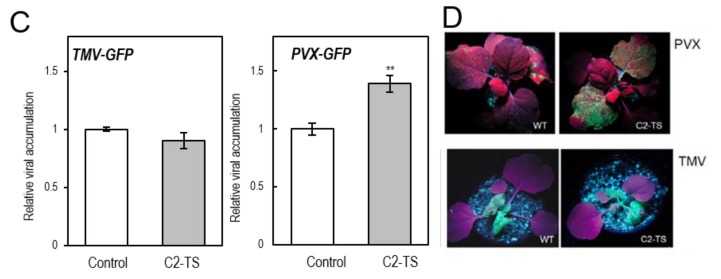
Transgenic C2-TS plants are more susceptible to *Pseudomonas syringae* pv. *tomato* DC3000 and *Potato virus X*. (**A**) Bacterial growth of *Pto* DC3000 in wild-type or C2-expressing Arabidopsis plants upon inoculation by infiltration. Samples were taken at 4 dpi. Values are the mean of five plants. Bars represent standard error. The asterisk indicates a statistically significant difference from the control sample (* *p*-value < 0.05) according to a Student’s *t*-test. Three independent experiments were performed with similar results; results from one representative experiments are shown; (**B**) Bacterial growth of wild-type *Pto* DC3000, a Δ*hrcC* mutant, or a wild-type strain expressing the heterologous effector AvrRpt2 on wild-type or C2-TS-expressing Arabidopsis plants. Values represent the average of five plants. Bars represent standard error. The asterisk indicates a statistically significant difference from the control sample (* *p*-value < 0.05) according to a Student’s *t*-test. Three independent experiments were performed with similar results; results from one representative experiments are shown; (**C**) Infection of wild-type (WT) or C2-TS-expressing *N. benthamiana* plants with PVX-GFP or TMV-GFP at 10 dpi. Values represent relative expression of viral RNA estimated by semi-quantitative RT-PCR, and are the average of ten infected plants. Bars represent standard error. Asterisks indicate a statistically significant difference from the control sample (** *p*-value < 0.01) according to a Student’s *t*-test. Two independent experiments were performed with similar results; results from one representative experiment are shown; (**D**) Pictures of representative TMV-GFP and PVX-GFP infected plants under UV light.

Based on the finding that defence responses are repressed in C2-TS plants (Figure 3; Table 2 and Table 3), we decided to test the susceptibility of C2-expressing transgenic lines to different pathogens. For this purpose, we infected transgenic plants expressing C2 with *P. syringae* or RNA viruses. *Arabidopsis* C2 plants were inoculated by infiltration with wild-type *Pto* DC3000, a Δ*hrcC* non-pathogenic mutant strain, or a wild-type strain expressing the heterologous effector AvrRpt2, which triggers an additional level of defences, the hypersensitive response (HR) [43]. Consistently with the transcriptional repression of the defence response, C2 plants are more susceptible to wild-type *Pto* DC3000 than wild-type plants when bacteria are infiltrated into the leaves (bypassing bacterial entry into the plant tissues) (Figure 4A), although no significant differences were found after infiltration with non-pathogenic or avirulent bacteria (Figure 4B).

To test the susceptibility to RNA viruses we agroinfiltrated *Nicotiana benthamiana* plants expressing C2 from TYLCSV (C2-TS plants), which also show reduced responses to exogenous MeJA [4], with infectious clones of *Potato virus X* (PVX) and *Tobacco mosaic virus* (TMV) labelled with GFP. C2-TS *N. benthamiana* plants were more susceptible to infection with PVX: the levels of GFP and the viral RNA expression in C2-TS plants were higher than in control plants when measured by semi-quantitative PCR (Figure 4C,D). However, no significant changes in viral accumulation were detected in plants inoculated with TMV-GFP (Figure 4). Altogether, these results suggest that C2 might be suppressing JA-induced basal defence responses.

## 3. Discussion

The C2 protein from geminiviruses, including the begomoviruses TYLCSV and TYLCV, has been shown to hinder the function of the SCF E3 ubiquitin ligases in the plant cell, possibly through its interaction with CSN5, catalytic subunit of the SCF regulator CSN [4]. Whereas this effect of C2 has been observed for several SCF complexes regulating diverse hormonal responses, the most pronounced transcriptional change resulting from transgenic expression of C2 in *Arabidopsis* is a suppression of the jasmonate response [4]. Since jasmonate signalling initiates with perception of the hormone by the SCF^COI1^ complex, it is feasible to speculate that partial inhibition of this E3 by C2 could underlie the detected suppression of this response. In agreement with this, *Arabidopsis* plants expressing C2 are not only less sensitive to jasmonates, but also to the bacterial JA-Ile mimic coronatine, also perceived by the SCF^COI1^ complex (Figure A1 and Figure 1). However, why the effect of C2 is more noticeable on the SCF^COI1^ than on other SCF complexes, and what the molecular mechanisms underlying this specificity are, is unclear.

In addition, it is remarkable that C2 from TYLCSV does not generally affect JA-induced transcription, and on the contrary seems to suppress specific responses, namely JA-induced defences and secondary metabolism (Table 1, Table 2 and Table 3; Figure 3). Again, the lack of a general effect on JA responses supports the idea of additional mechanisms acting superimposed to the effect on the jasmonate receptor, possibly relying on additional protein-protein interactions with downstream signalling components. Promoter analysis of the subsets of JA-responsive genes affected by C2, contained in Table 1 and Table 3, indicates a common over-representation of MYC- and MYC2-binding sites (data not shown), which suggests that C2 could be directly or indirectly interfering with the function of this family of transcription factors.

As expected from the transcriptional suppression of defence responses, C2-expressing *Arabidopsis* plants are more susceptible to the plant pathogenic bacterial strain *Pto* DC3000 when inoculated by infiltration (bypassing entry via stomata). This enhanced susceptibility, however, argues against an impairment of the apoplastic effect of coronatine. However, we cannot rule out the possibility that C2 may have additional targets impacting the interaction between the plant and *P. syringae*. Interestingly, since no differences can be detected in inoculations with the non-pathogenic mutant Δ*hrcC* or the strain expressing the HR-inducing effector AvrRpt2, C2 seems to be suppressing basal defence responses specifically. Additionally, *N. benthamiana* plants expressing C2 are more susceptible to the RNA virus PVX. JA-induced terpenoids have been recently shown to play a role in anti-viral defence against PVX [28] and, therefore, the C2-mediated suppression of JA could underlie the enhanced viral performance. No differences could be observed, however, in infections with the RNA virus TMV; these results are in agreement with the previous finding that jasmonate signalling does not affect susceptibility to this virus [44], although, strikingly, it seems to be required for systemic resistance [32].

Although the observation that C2 suppresses the jasmonate response has been well documented, a series of questions remain open. For example, the potential additional molecular mechanism conferring specificity in the suppression of this response is still elusive. Importantly, it also remains to be determined whether the observed effect in *Arabidopsis* also occurs in tomato, which is the virus” natural host; and, assuming this is the case, what the biological effect on both the virus itself and its insect vector is. Future work will be required to gain further understanding of this function of the viral C2 protein, and to get a comprehensive picture of its relevance for plant-virus-insect interactions.

## 4. Experimental Section

### 4.1. Plant Material and Growth Conditions

Wild-type *Arabidopsis thaliana* used in this study is the Columbia ecotype. Seeds were surface-sterilized and sown on MS agar plates with 30 g/L sucrose. Plates were cold-treated for 2 to 6 days at 4 °C. Seedlings were grown at 20 °C under fluorescent white light (fluence rate of 40–60 μmol·m^−2^·s^−1^) with a 16 h light/8 h dark photoperiod. For root growth inhibition assays, MS plates were placed in a vertical orientation for five days, and seedlings were then transferred to MS plates containing the 50 μM MeJA. Root length was scanned five days later using the ImageJ software (http://rsb.info.nih.gov/ij).

The transgenic *Arabidopsis* plants expressing C2 from TYLCSV and TYLCV and the transgenic *Nicotiana benthamiana* plants expressing TYLCSV C2 are described elsewhere [4].

For transcriptomic analysis, T2 seedlings of C2-TS transgenic *Arabidopsis* plants were grown on MS with kanamycin for seven days, and then treated with 50 μM MeJA or mock solution for 10 h. Three independent replicates were performed. For these analyses, T3 homozygous *LUC2* (*PRB1::LUC*) transgenic plants [45] resistant to kanamycin were used as control. Previously, the hormonal response of LUC2 had been proven to be identical to that of the wild-type in the aforementioned assays.

*N. benthamiana* plants were grown in soil at 22 °C in long day conditions (16 h light/8 h dark photoperiod).

### 4.2. Bacterial Infections

*Pseudomonas syringae* pv. *tomato* DC3000 (Cuppels, 1986), a mutant strain unable to produce coronatine (CFA^−^ CMA^−^; [46]), a Δ*hrcC* mutant [47], or a bacterial strain expressing the heterologous effector AvrRpt2 [48] were grown at 28 °C in LB medium supplemented with rifampicin (15 μg/mL) and kanamycin (15 μg/mL; in the case of *Pto* AvrRpt2). Bacteria were suspended in 10 mM MgCl_2_ before inoculations. Four to five-week old *Arabidopsis* plants were either inoculated by infiltrating with a 5 × 10^4^ cfu/mL bacterial suspension using a blunt syringe, or inoculated by dipping for 30 s in a 5 × 10^7^ cfu/mL bacterial suspension containing 0.02% silwet L-77 (Crompton Europe LTD, Evesham, UK). Symptoms were evaluated at 4 dpi. Samples were taken from inoculated leaves at 4 dpi using a 10 mm-diameter cork borer. Three disks were taken per plant, placed into 1 mL of 10 mM MgCl_2_, and homogenized by mechanical disruption. Serial dilutions of the resulting bacterial suspensions were plated onto LB plates supplemented with of cycloheximide (2 μg/mL) and rifampicin (15 μg/mL).

### 4.3. RNA Extraction, cRNA Preparation, and Affymetrix GeneChip^®^ Hybridization

The generation of these data has been described previously [49]. In brief, seven-day-old LUC2 and transgenic C2-TS *Arabidopsis* seedlings were treated with a 50 M MeJA o mock solution for 10 h. Three biological and three technical replicates were used. Total RNA was isolated from three replicates of MeJA- or mock-treated wild-type and transgenic C2-TS seedlings using TRIzol (Invitrogen, Carlsbad, CA, USA) and subsequently cleaned using RNeasy MinElute Cleanup Kit (Qiagen, Hilden, Germany). RNA quantity and quality were assessed with a Nanodrop ND-1000 spectrophotometer (Labtech, Ringmer, East Sussex, UK) and an Agilent 2100 bioanalyzer (Agilent Technologies, Santa Clara, CA, USA), respectively.

Microarray hybridization was carried out at the Unité de Recherche en Génomique Végétale (Evry, France), using Affymetrix GeneChip® ATH1.

All raw and normalized transcriptomic data are available through the CATdb database (project AFFY_MeJA_Arabidopsis) and from the Gene Expression Omnibus (GEO) repository at the National Center for Biotechnology Information (NCBI), under accession number GSE18667.

### 4.4. Viral Infections

*Tobacco mosaic virus* (TMV)-GFP and *Potato virus X* (PVX)-GFP are described elsewhere [50]. Infections in wild-type and transgenic C2-TS *N*. *benthamiana* were performed by agroinoculation as described in [50]. GFP expression was monitored at seven and 10 days post-inoculation (dpi), and samples were taken at 10 dpi.

### 4.5. Quantitative Real-Time PCR (qPCR)

Primer pairs for real-time PCR were designed using Primer 3 software (http://frodo.wi.mit.edu/primer3/). Gene-specific primers were chosen so that the PCR products were 100–300 bp. Total RNA was extracted from seedlings using RNAeasy Plant Mini Kit (Qiagen, Hilden, Germany) and treated on column with Dnase (Qiagen). 1 μg total RNA was used for first-strand cDNA synthesis using oligo(dT) primers and SuperScript II reverse transcriptase reagent (Invitrogen) following the manufacturer’s instructions. For real-time PCR, the reaction mixture consisted of cDNA first-strand template, primer mix (5 μmol each) and SsoFast™EvaGreen_®_Supermix (BIO-RAD, Hercules, CA, USA) in a total volume of 25 μL. The PCR conditions were: 10 min at 95 °C, and 40 cycles of 30 s at 95 °C and 30 s at 60 °C. The reactions were performed using a Rotor-Gene real-time cycler (Qiagen). A relative quantification real-time PCR method was used to compare expression of the genes in transgenic *versus* non-transgenic line [51]. Relative quantification describes the change in expression of the target gene in a test sample relative to calibrator sample. Actin was used as the internal control. The sample of LUC2 transgenic plants was used as the calibrator, with the expression level of the sample set to one. Each data point is the mean value from three experimental replicate determinations. Three biological replicates were used.

For quantification of PXV-GFP and TMV-GFP, total RNA was extracted from the third leave of each infected *N. benthamiana* plant using RNAeasy Plant Mini Kit (Qiagen) and treated on column with Dnase (Qiagen). cDNA synthesis was performed as previously described. Virus GFP accumulation was assessed by semi-quantitative PCR using primers for *GFP* (Up-mGFP: AGTGGAGAGGGTGAAGGTGA; low-mGFP: AAAGGGCAGATTGTGTGGAC) and the following conditions: 94 °C, 30 s; 55 °C, 30 s; 72 °C, 40 s (22 cycles). Primers to amplify the 16S-23S rDNA *interspacer* (*ITS*) were used as control (ITS 25S fw: ATAACCGCATCAGGTCTCCA; ITS 25S Rv: CCGAAGTTACGGATCCATTT) using the same PCR conditions, 16 cycles. Bands were quantified using ImageJ software (http://rsb.info.nih.gov/ij).
